# The redox environment triggers conformational changes and aggregation of hIAPP in Type II Diabetes

**DOI:** 10.1038/srep44041

**Published:** 2017-03-13

**Authors:** Diana C. Rodriguez Camargo, Konstantinos Tripsianes, Katalin Buday, Andras Franko, Christoph Göbl, Christoph Hartlmüller, Riddhiman Sarkar, Michaela Aichler, Gabriele Mettenleiter, Michael Schulz, Annett Böddrich, Christian Erck, Henrik Martens, Axel Karl Walch, Tobias Madl, Erich E. Wanker, Marcus Conrad, Martin Hrabě de Angelis, Bernd Reif

**Affiliations:** 1 Helmholtz Zentrum München, Ingolstädter Landstr. 1, Neuherberg 85764, Germany; 2 Munich Center for Integrated Protein Science (CIPS-M) at Department Chemie, Technische Universität München (TUM), Germany; 3 Central European Institute of Technology (CEITEC), Masaryk University, Kamenice 5, Brno 62500, Czech Republic; 4 German Center for Diabetes Research (DZD e.V.), Neuherberg 85764, Germany; 5 Max-Delbrück-Center Berlin (MDC), Robert-Rössle-Str. 10, Berlin 13125, Germany; 6 Synaptic Systems GmbH, Rudolf-Wissell-Straße 28, Göttingen, 37079, Germany; 7 Institute of Molecular Biology & Biochemistry, Center of Molecular Medicine, Medical University of Graz, Austria; 8 Technische Universität München, Center of Life and Food Sciences Weihenstephan, Freising 85354, Germany

## Abstract

Type II diabetes (T2D) is characterized by diminished insulin production and resistance of cells to insulin. Among others, endoplasmic reticulum (ER) stress is a principal factor contributing to T2D and induces a shift towards a more reducing cellular environment. At the same time, peripheral insulin resistance triggers the over-production of regulatory hormones such as insulin and human islet amyloid polypeptide (hIAPP). We show that the differential aggregation of reduced and oxidized hIAPP assists to maintain the redox equilibrium by restoring redox equivalents. Aggregation thus induces redox balancing which can assist initially to counteract ER stress. Failure of the protein degradation machinery might finally result in β-cell disruption and cell death. We further present a structural characterization of hIAPP in solution, demonstrating that the N-terminus of the oxidized peptide has a high propensity to form an α-helical structure which is lacking in the reduced state of hIAPP. In healthy cells, this residual structure prevents the conversion into amyloidogenic aggregates.

Type II diabetes (T2D) is the most common form of diabetes. The disease is caused by a combination of genetic and environmental factors, and results in insulin resistance and β-cell failure^1^. T2D is also linked to the tissue inflammation response^2^. The exact patho-mechanism of the disease is, however, not understood so far. In addition to insulin resistance, lipotoxicity, endoplasmic reticulum (ER) and oxidative stress, as well as amyloid deposition have been described as secondary effects of the disease^2^. The amyloid plaques formed within the β-cells of the pancreatic islets of Langerhans^3^ are composed of the 37 amino acid polypeptide human Islet Amyloid Polypeptide (hIAPP). In recent studies it has been shown that soluble oligomers of this hormone are responsible for cell toxicity^4^,^5^. Yet until now, it is not understood how hIAPP changes from a functional into a pathophysiological form. *In vivo*, hIAPP is soluble at concentrations of 1–4 mM^6^,^7^,^8^, while *in vitro* hIAPP aggregates at 1000 times lower concentrations. Protein aggregation is influenced by concentration and pH^9^. In T2D, the redox state of the ER is shifted towards more reducing conditions, resulting in protein secretion and folding deficiencies^10^,^11^,^12^. So far, it is unclear how this change of the redox environment affects hIAPP aggregation. We show here that the redox conditions play an important role in the aggregation of hIAPP. Furthermore, we present the first detailed structural analysis of monomeric hIAPP in aqueous buffer. We find that the disulfide bridge in hIAPP^ox^ stabilizes an α-helical structure at the N-terminus of the peptide, and thus protects the peptide from aggregation.

## Results

### Assembly Kinetics of hIAPP

**Figure 1:**
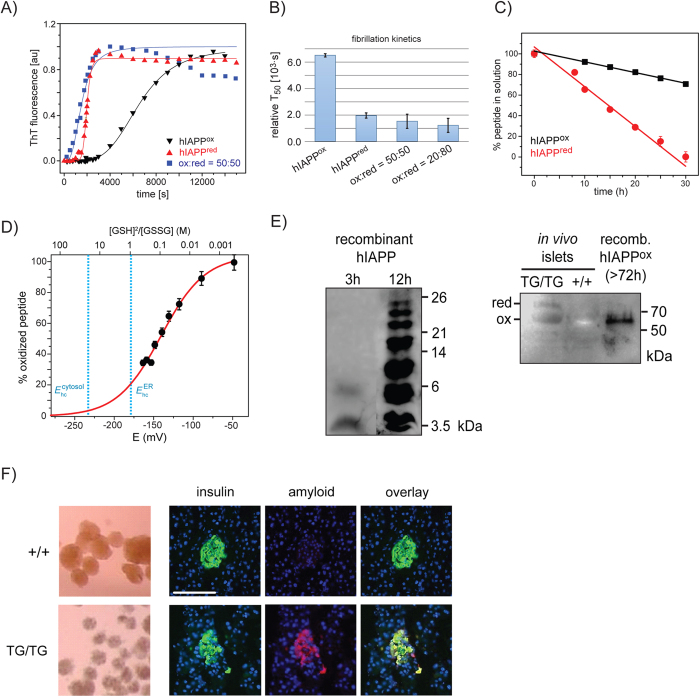
(**A**) Thioflavin T (ThT) assay using a solution of 35 μM of hIAPP, pH 5.3 and 30 °C. Experiments were performed by adding defined amounts of GSH and GSSG to yield varying percentages of reduced and oxidized hIAPP in solution. In all three curves, ThT fluorescence intensities are normalized to 1. ThT fluoresence curves with absolute intensities are represented in Fig. S2. (**B**) T_50_ values for hIAPP aggregation for different redox conditions. T_50_ corresponds to the time that is required to reach half-maximum ThT fluoresence intensity. (**C**) Aggregation kinetics of hIAPP^ox^ and hIAPP^red^ measured by solution-state NMR. hIAPP^red^ aggregates significantly faster in comparison to hIAPP^ox^. The intensities of the NMR resonances directly indicate the amount of soluble hIAPP^ox^ and hIAPP^red^. Both peptides were solubilized at a concentation of 100 μM in aqueous buffer (30 mM acetic acid, pH 5.3, 4 °C) (**D**) Determination of the electrochemical potential of hIAPP. The relative amount of hIAPP^ox^ is represented as a function of the concentrations of GSH and GSSG. For the analysis, the peak intensity of Cys-7 has been employed. The redox potential of hIAPP was determined as −129 ± 4 mV using the protocol described by Zimmermann *et al*.^16^. (**E**) Western blot of *in vitro* and *in vivo* formed hIAPP aggregates. Left: Purified, recombinant hIAPP after an incubation time of 3 h and 12 h in solution yields SDS-stable hIAPP oligomers. The oligomeric state is increased at prolonged incubation times. Right: Sample extracted from pancreatic islets from diabetic TG/TG and non-diabetic+/+ mice. The oxidation state of cysteines was blocked with NEM in an alkylation reaction which produced a modified hIAPP^red^ exhibiting a larger molecular weight. The high molecular weight bands were observed only in the TG/TG mouse sample. hIAPP was identified using the antibody A133^15^. This antibody is monoclonal against hIAPP(20–29) and is specific for the human sequence. No interference with alkylated hIAPP can thus be expected. (**F**) Light microscopy (left) and histological immunofluorescence images (right) of islets from +/+ control mice and TG/TG mice. Insulin is indicated in green, amyloid fibrils in red, nuclei in blue. +/+ control mice do not display any morphological changes. Amyloid aggregates are observed using an antibody against amyloid fibrils in islets of TG/TG mice. The white scale bar denotes 100 μm. Figure S1 shows that the antibody 91D7E8 speciflcally detects IAPP fibrils.

To better understand the influence of the redox environment on the aggregation kinetics of hIAPP, we first performed Thioflavin T (ThT) assays. In a cell, the ratio of reduced glutathione (GSH) to oxidized glutathione (GSSG) is often employed as a measure of cellular oxidative stress. In order to induce a particular oxidative state of hIAPP, we added the redox couple GSH/GSSG to the peptide solution (Fig. 1A,B). A sigmoidal fit of the ThT results yields the time at which the half-maximum fluorescence intensity has been obtained (*T*_50_). We found that the aggregation kinetics of hIAPP are affected by the oxidative environment. hIAPP^red^ aggregates faster in comparison to hIAPP^ox^. However, mixtures of hIAPP^red^ and hIAPP^ox^ yield the same rate of aggregation as hIAPP^red^. Within the experimental error, we found the same *T*_50_ values for redox pairs that yield (100:0)%, (80:20)% or (50:50)% reduced and oxidized hIAPP. This suggests that the aggregation of hIAPP is controlled by the reduced form. The ThT results are supported by NMR experiments which show that hIAPP^red^ has a 3-times faster aggregation kinetics in comparison to hIAPP^ox^ (Fig. 1C). To validate the biological significance of the differential aggregation behaviour of hIAPP^ox^ and hIAPP^red^, we determined the redox potential for the formation of the disulfide bond in hIAPP using NMR spectroscopy. ^1^H,^15^N HSQC spectra were recorded after incubating the protein with varying ratios of reduced and oxidized glutathione. A plot of the average peak intensities as a function of the [GSH]^2^/[GSSG] ratio is shown in Fig. 1D. As a secondary abscissa, the calculated redox potential is represented. Using the Nernst equation with the respective correction for temperature and pH^13^, we calculated a redox potential of (−129 ± 4) mV for hIAPP. The ER has an electrochemical potential that varies between −231 to −160 mV, and is dependent on the employed method^14^. Under these conditions, less than 30% of the peptide exists in the oxidized state. This theoretical redox state assumes quasi-equilibration with the redox couple GSH/GSSG.

To analyze the hIAPP oxidation state *in vivo*, we used a transgenic mouse model (FVB/N-Tg(Ins2-IAPP)RHFSoel/J) in which the human IAPP sequence has been incorporated into the mouse genome on chromosome 15 (https://www.jax.org/strain/008232). We found that the morphology of the islets is dramatically changed for transgenic mice that harbor two human gene copies (TG/TG) (Fig. 1F, left) compared to healthy controls. We employed a fibril specific antibody to study whether amyloid fibrils participate in this process. We found amyloid fibrils in insulin producing β-cells in TG/TG animals (Fig. 1F). These were not present in healthy animals. These results suggest that amyloid fibrils in TG/TG mice potentially induce impaired islet morphology. To quantify the amount of reduced or oxidized hIAPP *in vivo*, we added N-ethylmaleimide (NEM) to TG/TG and +/+ mouse islets to yield an alkylation of free sulfhydryl groups. NEM has the ability to cross cellular membranes and thus does not require cell lysis. Modified hIAPP^red^-NEM yielded an increased molecular weight which could be detected by SDS-PAGE. Soluble oligomeric hIAPP could be identified using the hIAPP specific antibody A133^15^. In TG/TG islets, both reduced and oxidized hIAPP were found (Fig. 1E, right). As expected, no aggregates were found in the islets of the+/+control mice (Fig. 1E, right). As a reference, an aggregated sample of recombinant, oxidized hIAPP was investigated as well (Fig. 1E, left). We observed that samples that were taken at a later time point yielded a more disperse distribution of oligomeric states. An incubation time of 72 h for an oxidized hIAPP sample produced a gel band at approximately 60 kDa, which corresponds in molecular weight to the band of the hIAPP sample extracted from the diabetes mouse model islets (Fig. 1E, right, last sample).

**Figure 2:**
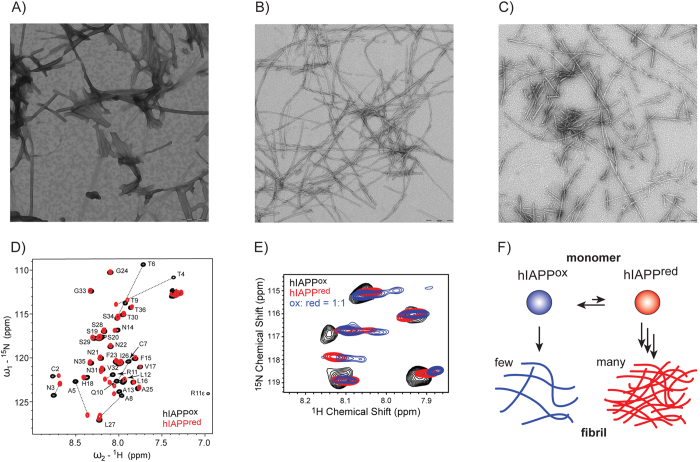
(**A–C**) EM images of fibrils formed in the presence of different ratios of the GSH-GSSG redox couple. In (**A**) hIAPP was incubated using 2 mM GSSG to produce a completely oxidized hIAPP. (**B**) hIAPP was incubated using 35 mM GSH yielding 100% hIAPP^red^, whereas in (**C**) hIAPP was incubated in a buffer containing 0.2 mM GSSG and 4.98 mM GSH yielding 50% hIAPP^red^ and 50% hIAPP^ox^. The scale bar denotes 200 nm. (**D**) ^1^H,^15^N-HSQC spectra of the reduced (red) and oxidized (black) hIAPP in aqueous buffer (30 mM acetic acid, pH 5.3). Amino acids are labelled according to their type and sequence number. Major chemical shift changes upon disulfide bond formation are indicated by black dashed lines. The NMR chemical shifts are deposited in the BMRB (BMRB ID: 34069). (**E**) ^1^H,^15^N HSQC spectra to probe the hIAPP redox state. Fibrils were prepared under the three redox conditions described in (**A–C**). Before the NMR experiment, fibrils were dissolved in organic solvent. (**F**) Model of the hIAPP aggregation mechanism. ER stress results in production of a small amount of hIAPP^red^ in the cell. hIAPP^red^ aggregates preferentially and is depleted from the pool of soluble molecules in the cell. Redox buffering continuously converts hIAPP^ox^ into hIAPP^red^. This way, hIAPP might assist in regenerating redox equivalents under ER stress conditions.

In order to probe if the differential aggregation behavior results in a continuous conversion of hIAPP^ox^ into hIAPP^red^ at defined redox conditions, electron microscopic (EM) images of hIAPP aggregates that are obtained in buffers with different amounts of GSH and GSSG were recorded (Fig. 2A–C). Under conditions, in which the redox equilibrium is shifted to produce a defined amount of hIAPP^ox^ (50:50 = hIAPP^red^:hIAPP^ox^) (Fig. 2C), the fibril morphology resembles the morphology obtained for the pure hIAPP^red^ (Fig. 2B). By contrast, pure hIAPP^ox^ (Fig. 2A) showed a different fibril morphology. To confirm this observation, we carried out solution-state HSQC NMR experiments of hIAPP aggregates that were dissolved in DMSO prior to the experiment. These experiments allow to probe the hIAPP redox state of the hIAPP aggregates. Again, we found that the hIAPP spectra of the samples in which the redox properties in the buffer has been adjusted to yield 100% hIAPP^red^ or a 1:1 ratio of hIAPP^ox^ and hIAPP^red^ are rather similar, whereas the sample containing pure hIAPP^ox^ looked significantly different (Fig. 2E). This indicates that aggregation in fact shifts the equilibrium to the reduced state of the peptide. Based on these results, we suggest a model for hIAPP aggregation in the cell that is dependent on its particular redox environment (Fig. 2F). External factors such as oxidative stress or ER stress, hyperglycemia, lipotoxity, or genetic factors have an impact on the ER redox state. A small shift of the redox conditions can result in generation of a small amount of hIAPP^red^. hIAPP^red^ preferentially aggregates and is depleted from the pool of hIAPP molecules in solution. As the redox state in the cell is buffered, more hIAPP^ox^ is transformed to its reduced form which again has a higher propensity to form fibrils. Eventually, all available hIAPP is converted into its reduced state which is finally deposited in insoluble aggregates.

### Structural analysis of hIAPP

**Figure 3:**
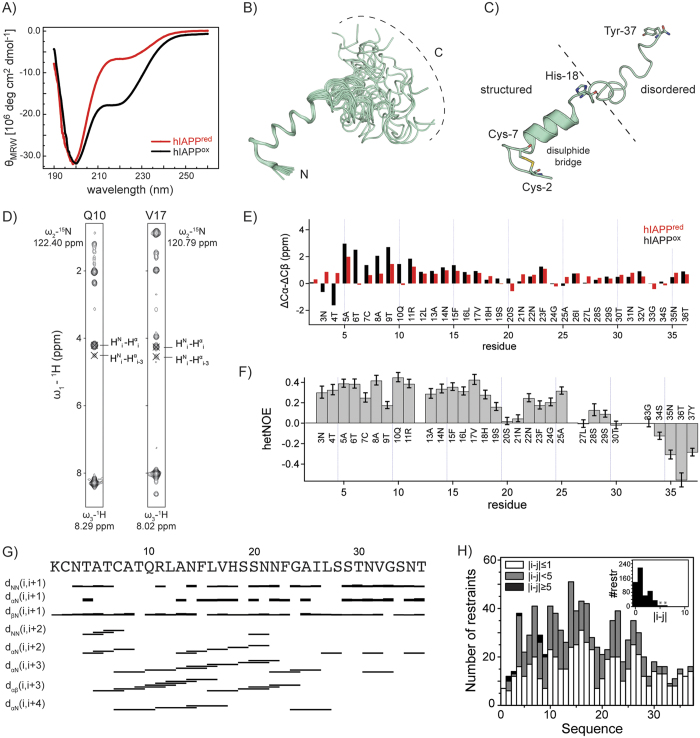
(**A**) Circular dichroism (CD) spectra of reduced (red) and oxidized hIAPP (black). hIAPP^ox^ yields a negative band at 222 nm and 208 nm which is characteristic for an α-helical conformation. The reduced form shows a considerably smaller band at 222 nm indicating a larger degree of random coil conformation. (**B**) NMR structural ensemble of hIAPP^ox^(1-37). The structure was determined at pH 5.3 and at a temperature of 4 °C, using the software package CYANA^21^. (**C**) Structure of one conformer of the hIAPP ensemble. hIAPP^ox^ adopts an α-helix structure involving residues 8–17, and is disordered in its C-terminal part. The disulfide bridge is indicated in yellow. The hIAPP structural ensemble was deposited in the PDB (PDB ID: 5MGQ). RDC experiments confirm that the N-terminal part of hIAPP adopts a compact structure (Fig. S4). (**D**) Representative strips from 3D NOESY experiments. The NOE pattern of the respective residues demarcates the boundaries of the α-helical structure of hIAPP. E) ^13^C^α/β^ secondary chemical shift analysis. Differences of deviations from random coil chemical shifts for Cα and Cβ (δΔ, ppm) are represented as a function of the primary sequence. hIAPP^red^ is shown in red. Reduction was induced by adding 10 mM TCEP. The oxidized peptide was obtained by addition of 2 mM H_2_O_2_ (black). Secondary Structure Propensity (SSP) of hIAPP^ox^ in aqueous buffer, pH 5.3, is shown in Fig. S3. (**F**) Heteronuclear NOEs of hIAPP. The hetNOE data confirms that the N-terminus of hIAPP has a high propensity to form a compact structure. (**G**) Experimental short and medium range NOE connectivities in hIAPP. (**H**) Number of NOE restraints per residue employed for hIAPP structure calculation. The inset displays the number of NOE restraints observed between residue i and j. Only four long range connectivities (residues i and j, with |i − j| > 5) are observed for the residues around the disulfide bridge (denoted with an asterisk in the inset).

To study the mechanism of hIAPP aggregation, the conformation of the peptide was determined under physiological conditions. Recombinant C-terminal amidated hIAPP(1-37) was produced using the protocol published recently^17^. hIAPP has a strong tendency to aggregate in aqueous buffer. However, low pH (pH 5.3), temperature (4 °C) and concentration (100 μM) reduced the rate of fibril formation. Under these conditions, hIAPP(1-37) yielded a CD spectrum which contained contributions from α-helix and random coil secondary structure (Fig. 3A). Comparison of the oxidized and the reduced form of the peptide suggests that a loss of the disulfide bond induces a loss of the α-helical structure. To support this observation, we recorded solution-state NMR experiments. ^1^H,^15^N HSQC spectra of reduced and oxidized hIAPP yielded significant chemical shift differences, in particular for Cys-2 and Cys-7 as well as for residues in the vicinity of the disulfide bond (Fig. 2D). Chemical shift assignments have been obtained from 3D HNCACB experiments^18^. The analysis of 13 C^α/β^ secondary chemical shifts reveals a strong helical propensity for the N-terminal half of the oxidized hIAPP (residues 5-17), and a random coil conformation for the C-terminal half of the peptide (Fig. 3E). The same analysis for the reduced form of hIAPP showed a smaller helical propensity for the N-terminal half, suggesting that the N-terminal disulfide bond stabilizes the helical conformation. The observation that hIAPP^ox^ has a diminished tendency to aggregate stimulated a more detailed structural characterization of the peptide. For the structural analysis, 3D ^13^C/^15^N edited NOESY-HSQC data were collected for hIAPP^ox^. The faster aggregation kinetics of hIAPP^red^ prohibited a detailed structural characterization. The ensemble of the 20 lowest energy structures obtained after water refinement is shown in Fig. 3B,C. A summary of the structural and restraint statistics is given in Table 1. We note that no hydrogen bonding restraints were included in the structure calculations to enforce an α-helical conformation. The present solution structure of native hIAPP (oxidized and amidated) shares similarities, but also has differences when compared to other structures of amylin reported under various conditions^19^. A common theme in all structures is the N-terminal loop closed by the disulfide bond followed by a right-handed α-helix. In our study, the oxidation state of the peptide was confirmed by the ^13^Cβ secondary chemical shifts of the two cysteines (Cys-2 and Cys-7). In our analysis, the α-helix spans residues 8–17. In particular, the helical structure was confirmed by the characteristic αN(i,i+3), αN(i,i+4) and αβ(i,i+3) NOEs (Fig. 3D,G,H). We found that the histidine at position 18 is incompatible with an α-helical conformation. His-18 thus introduces a kink in line with earlier findings (Fig. 3C)^20^. For the C-terminal part, the NOE pattern of the ^15^N-edited 3D NOESY experiment was dominated by strong correlations between the respective amides and the (i−1) Hα atoms which is typical for IDPs (Intrinsically Disordered Proteins). In addition, no long-range NOEs were detected for the C-terminal amide suggesting that cross-talk to distant residues is not occuring. The structural model is supported by {^1^H},^15^N heteronuclear NOE data that showed a certain rigidity for the N-terminal half and increased flexibility towards the C-terminus of hIAPP (Fig. 3F).

## Discussion

Stabilization of the monomeric state of a protein can prevent its conversion into an aggregated state^22^. This paradigm is generally accepted for folded proteins, but has so far not been recognized for disordered peptides and proteins. We show here that the oxidized form of the islet hormone hIAPP is more structured in comparison to the reduced form of the peptide, and at the same time has a smaller propensity to aggregate. We find furthermore that the pancreatic islets of a T2D mouse model contain primarily aggregated hIAPP in its reduced state. We suggest that differential aggregation unleashes redox equivalents that potentially counteract oxidative stress.

Initially, it was suggested that hIAPP adopts a random coil structure^23^. However, more recently reports indicate that the N-terminal part of hIAPP involving residues 5–19 and to a somewhat smaller degree residues 20–22 transiently samples an α-helical structure in solution^8^,^24^. The same residues adopt a fully helical structure when bound to membranes or micelles^19^,^24^,^25^. In agreement with previous studies, we found that reduction of the N-terminal disulfide bond (Cys2–Cys7) in solution decreases the extent of helix formation in this region^26^. Miranker and coworkers observed that formation of helical structure is directly correlated with enhanced amyloid formation on a membrane surface. It was suggested that a parallel association of helices results in locally increased peptide concentration that could nucleate β-strand structure. By contrast, we find that the reduced form of hIAPP aggregates significantly faster than the oxidized peptide. Our data can be reconciled with the observation made by Miranker and coworkers by assuming that membranes can favor an α-helical structure e.g. by formation of an amphipatic helix. At the same time, an increase of the local protein density can be a very strong driving force for aggregation. In aqueous buffer, the situation is fundamentally different. The α-helical propensity at the N-terminus of hIAPP impedes conversion into β-sheet structure. Solid-state NMR studies showed that hIAPP adopts a strand–turn–strand structure in amyloid fibrils, with the N-terminal and C-terminal strands involving residues 8–17 and residues 28–37, respectively^27^,^28^. The residues in-between the disulfide bridge do not contribute to the amyloid fiber core structure. At the same time, the disulfide bridge plays a central role in the fibril assembly mechanism^29^: Loss of the disulfide bond substantially reduces fiber formation by secondary nucleation, and eliminates the activation step in seeded fiber growth^30^.

Obviously, the expression level of hIAPP has a strong impact on solubility and aggregation propensity of the peptide. Insulin and hIAPP tend to be regulated in parallel. Hyperglycemia, however, has been shown to result in disproportionate upregulation of hIAPP versus insulin, thus altering the ratio of hIAPP to insulin secreted by the β-cells^31^. Increased secretion of hIAPP in severe hyperglycemia has been suggested to facilitate the formation of hIAPP-derived islet amyloid deposits^31^. At the same time, disturbances in redox regulation, calcium homeostasis, glucose deprivation, and viral infection can lead to ER stress^32^. Under these conditions, the folding of proteins is slowed down, leading to an increase of unfolded proteins. Unfolded proteins cause a stress response in the ER which is known as unfolded protein response (UPR)^33^. ER stress is emerging as a potential cause of damage in hypoxia/ischemia, insulin resistance, and other disorders^34^. In T2D, pancreatic β-cells undergo cell death as a consequence of sustained ER stress^35^,^36^. We find that reduced hIAPP aggregates 3-times faster in comparison to the oxidized form of the peptide. This differential aggregation results in a continuous conversion of hIAPP^ox^ into hIAPP^red^ in the ER. Thus, oxidation equivalents are continuously restored in the ER, a mechanism that might aid to counteract ER stress. Given the fact that hIAPP occurs at concentrations up to 4 mM in the cell^37^,^38^, hIAPP might act as a buffering system. This process works as long as the resulting aggregates are efficiently removed. Clearance can be achieved via autophagy and degradation in the lysosome. It has been shown that β-cell apoptosis is in fact correlated with autophagy and lysosomal degradation^39^,^40^,^41^. Inhibition of autophagy induces apoptosis, whereas stimulation of autophagy protects β-cells from hIAPP-induced toxicity. Alternatively, the endoplasmic reticulum–associated degradation (ERAD) assists to protect cells from ER stress^42^. It has been reported that accumulation of polyubiquitinated proteins occurs in pancreatic islets of hIAPP transgenic mice but not in mice with comparable transgenic expression of rodent-IAPP (r-IAPP). Proteasome dysfunction can mediate obesity induced ER stress and insulin resistance^43^. In fact, it has been found that the β-cell ERAD/ubiquitin/proteasome system of obese individuals with high BMI, that are otherwise healthy and not suffering from T2D, adapts to the increased synthetic burden of insulin and IAPP^44^. p62, an important factor in the autophagy/lysosomal pathway, is downregulated by overproduction of hIAPP in diabetic β-cells, resulting in induction of apoptosis^39^,^45^.

By contrast, the percentage of β-cells with ubiquitin-positive immunostaining was significantly increased in obese individuals with T2D. This may suggest that misregulation of proteasomal degradation yields an accumulation of aggregates which may eventually lead to perturbations in redox balance. *In vivo*, expression of other factors such as BiP, HSP70 and DnaJ which are known to interact with hIAPP can modulate the aggregation behaviour^46^. Similar to the small heat shock proteins such as αB-crystallin that interacts sub-stoichiometrically with Alzheimer’s disease Aβ^47^, HSP70 and GRP78/BiP can detect and bind misfolded hIAPP oligomers. Chaperones thus would have an influence ensuring proper redox balance.

Recently, it has been shown that the redox status of the microsomal protein folding machinery is affected in diabetes^48^. Streptozotocin-induced diabetes in rats results in a significant increase in the content of free protein sulfhydryl groups, as well as in the amount of free sulfhydryl groups of the major protein disulfide isomerases (PDIs), in particular the 58 kDa PDI and the 57 kDa ERp57. In this chemically induced diabetes model, hIAPP is overexpressed relative to insulin^49^. Our data revealed the redox potential of hIAPP to be on the order of (−129 ± 4) mV suggesting that a significant amount of hIAPP in the ER actually exists in the reduced state. A shift of the redox conditions under ER stress to a more reducing state, and an increase in the amount of produced hIAPP in T2D would immediately result in enhanced hIAPP aggregation. As a consequence of aggregation, even more redox equivalents would become available to restore the oxidizing redox environment of the ER.

Taken together, we have shows that aggregation of hIAPP can have beneficial effects on the well-being of the cell by restoring redox equivalents in the due course of aggregation. Only failure of the preoteasome degradation machinery results in β-cell disruption. It remains to be seen which molecular mechanisms in more detail induce a misfunction of proteasomal degradation in T2D.

## Materials and Methods

### Recombinant protein expression and purification

Human IAPP was expressed in *E. coli* and purified using the protocol described previously^17^. This protocol allows the production of an isotopically enriched peptide, which is amidated at the C-terminus and is disulfide bridged involving residues Cys-2 and Cys-7. Molecular biology reagents were obtained from Roche, New England Biolabs and from Sigma-Aldrich St. Louis, MO, USA. Isotopically labeled minimal media components were purchased from Cambridge Isotope Laboratories (CIL).

### Isolation of mouse islets

hIAPP transgenic mice were purchased from Jackson Laboratory (Bar Harbor, USA) and studied between 13–18 weeks of age. Animals were killed by isoflurane overdose and islets were isolated immediately. To perfuse the pancreas, the common bile duct was clamped at the papilla of Vater and 1 mg/ml cold collagenase P solution in HBSS solution supplemented with 1% BSA was injected into the common bile duct. The pancreas was removed and incubated for 15 min at 37 °C. Digestion was stopped by addition of a cold HBSS solution supplemented with 1% BSA and centrifuged at 290 g for 2 min at 4 °C. The pellet was resuspended in the HBSS solution supplemented with 1% BSA, filtered through a metal mesh and centrifuged at 290 g for 2 min at 4 °C. The pellet was resuspended in 15% Optiprep supplemented with 3.75 mM HEPES, 12% PBS, 6.2% RPMI and layered onto 15% Optiprep supplemented with 3.75 mM HEPES, 12% PBS, 6.2% RPMI solution. The pancreatic lysate was overlaid with the HBSS solution supplemented with 1% BSA, incubated for 10 min and centrifuged at 360 g for 18 min at 4 °C. After centrifugation, islets were observed in the interface between the first and second layers and after collecting islet fraction, cell debris were filtered through a 70 μm cell strainer and islets were washed from the filter with RPMI supplemented with 11 mM glucose, 10% FBS, antibiotic-antimycotic solution. To eliminate remaining exocrine tissue, the islets were handpicked and transferred into a new culture dish.

Animals were declared to the responsible local authority (Regierung von Oberbayern). The Institutional Animal Welfare Officer (Helmholtz-Zentrum München) was consulted about the work and approved the study. All experiments were performed in accordance with relevant guidelines and regulations.

### Western Blot Analysis

Extracted islets were solubilized with 30 μL PBS buffer supplemented with NEM and vortexed. Subsequently, 10 μL of SDS sample buffer without reducing agent was added. Samples were boiled for 5 min at 90 °C and separated by 12% Mini-PROTEAN-TGX stain-Free^TM^ protein gels, and then transferred onto a Tnas-Blot-Turbo^TM^ Mini PVDF membrane with a Bio-Rad Trans-Blot Turbo^TM^ transfer system. Membrane was blocked in Tween-Tris-buffered saline with 5% (w/v) nonfat dry milk (Roth) for 1 hour and then incubated with the primary antibody A133 in Tween-Tris-buffered saline with a ratio 1:1000 overnight at 4 °C. Reactivity was analyzed with antirabbit peroxidase-conjugated secondary antibody (Serva) and chemiluminescence detection (Bio-Rad).

### hIAPP fibril antibody production

To generate a monoclonal antibody against hIAPP amyloid fibrils, synthetic human lyophilized HFIP-treated hIAPP was dissolved in 20 mM Tris buffer (pH 7.4) to a concentration of 30 μM. The solution was sonicated for 1 min and incubated for 2 d at 37 °C with shaking at 300 rpm. The resulting fibrils were used for antibody generation according to a proprietary immunization protocol of Synaptic Systems Göttingen (see also http://www.sysy.com/mabservice.html): Three 8–10-week-old BALB/c female mice were subcutaneously immunized with IAPP fibrils over a period of 17 days. Cells from the knee lymph nodes were fused with the mouse myeloma cell line P3 × 63Ag8.653 (ATCC CRL-1580). The clones used in this study were re-cloned two times by limiting dilution and the immunoglobulin subclass was determined. Antibodies secreted by the hybridomas were screened for their reactivity against the immunogen using ELISA. Positive antibodies were retested by ELISA against hIAPP fibrils and monomer. Hybridomas producing antibodies with preference for IAPP fibrils were subcloned to monoclonality and further analyzed for histology and western blot applications. One of the antibodies showing high specificty for hIAPP fibrils was the antibody 91D7E8.

### Immunofluorescence staining of pancreatic β-cells

Pancreata were fixed in 4% paraformaldehyde and processed as published previously^50^. Briefly, cryo-sections were stained with anti-insulin (Invitrogen, Life Technologies, Germany), or with anti-amyloid fibril antibody 91D7E8 employing corresponding fluorescent-labeled secondary antibodies (Invitrogen, Life Technologies, Germany). DAPI was used to visualize cell nuclei. Slides were scanned using a NanoZoomer 2.0 HT (Hamamatsu, Japan) fluorescence scanner.

### NMR sample preparation

Lyophilized hIAPP(1-37) was dissolved into NMR buffer (30 mM deuterated acetic acid, pH 5.3) containing 10% D_2_O for locking. The samples were measured and stored at 4 °C. The formation of the intramolecular disulfide bond was confirmed by NMR. To prepare hIAPP in the oxidized state, a hIAPP solution was supplemented with 0.2 mM GSSG. To generate the reduced form of the peptide, the GSH powder was directly added to the hIAPP solution to yield a final concentration of 35 mM of reduced glutathione. To produce the hIAPP^red^:hIAPP^ox^ = 50:50 mixed sample, the hIAPP stock solution was added to a glutathione solution with final concentrations of 0.2 mM GSSG and 4.98 mM GSH. The final hIAPP concentration amounted to 100 μM in all cases. Afterwards, the samples were incubated at 4 °C to allow for complete oxidation or reduction.

For analysis of the oxidation state of the aggregated hIAPP samples at varying redox conditions, peptides were incubated first for two weeks at 37 °C to allow for fibril formation. After this period, samples were centrifuged for 30 min to harvest the fibrils. Pellets were dissolved in 250 μL of d_6_-DMSO, and subsequently sonicated for 1 hour. In order to prevent degradation of hIAPP, ice was added into the sonication bath to avoid sample heating. Afterwards, samples were directly transferred into Shigemi NMR tubes (Shigemi Inc., Allison Park, USA) for solution-state NMR measurements.

### NMR Experiments

All NMR experiments were performed at 4 °C, employing Bruker Avance 500, 600, 750 MHz spectrometers, equipped with cryo-probes. The proton chemical shifts were referenced to the water resonance frequency, the ^15^N and ^13^C shifts were referenced indirectly. Backbone and side chain assignments were obtained using triple resonance experiments^51^. Side chain assignments were obtained from 3D-HCCH TOCSY experiments. Overall, an assignment completeness of 97% was obtained. NMR spectra were processed using the software TopSpin (Bruker) and NMRPIPE^52^. Spectra were analyzed using SPARKY^53^ and ccpNMR analysis^54^.

### Structure Calculation

Sequential and inter-residual NOE cross-peaks that could be resolved in the ^15^N-edited spectrum were assigned manually for the N-terminal half and were kept fixed during structure calculations with CYANA^21^. Peak intensities for all cross-peaks were converted to distances using the internal function of CYANA. Dihedral angle restraints were derived from chemical shifts using the program TALOS^55^,^56^. Distance and angle restraints were used as input for the calculation of the structure on a linux-based cluster. All non-violated structures were recalculated using a water-refinement protocol.

### Transmission electron microscopy (TEM)

Samples were stained with 1% uranyl acetate solution on Formvar/Carbon 300 mesh copper coated grids from Electron Microscopy Sciences (Hatfield, PA 19440, USA). 10 μL of the sample were placed on the grid for 1 min. The excess of the sample was dried using filter paper. After this process, the grid was washed by placing it three times on a drop of water for 3 s. For staining, the grid was placed on a drop of a uranyl acetate solution for 30 s. The excess of the solution was again dried using filter paper. Samples were measured immediately employing an EM 10 CR (Zeiss, Germany).

### Determination of the hIAPP redox potential

100 μM hIAPP^ox^ was initially dissolved in 500 μL NMR buffer, containing 0.2 mM oxidized glutathione (GSSG). The GSH concentration was then increased stepwise to yield a final GSH concentration of 0.2, 1.0, 3.0, 5.0, 7.0, 10.0, 12.0, 15.0 and 18.0 mM, using a 100 mM GSH stock solution. ^1^H,^15^N-HSQC spectra were recorded after each addition of GSH. To determine the redox potential, the intensity of Cys-7 was determined in each spectrum. The intensities of the Cys-7 cross peak were plotted as a function of [GSH]^2^/[GSSG] on a logarithmic scale, and fit assuming a sigmoidal behavior. The [GSH]^2^/[GSSG] titration half-point was determined as 0.24 M. The redox potential for the GSH/GSSG redox pair was assumed to be −151 mV^13^. The Nernst equation yields then a redox potential for hIAPP of −129+/−4 mV.

### Circular Dichroism Spectroscopy (CD)

CD experiments were carried out at room temperature employing a JASCO spectropolarimeter. Quartz cells with a path length of 0.1 cm were used. Spectra were recorded between 260 nm and 190 nm at 0.1 nm intervals, with a response time of 1 s. For the analysis of the data the background buffer was subtracted and the data were expressed as mean residue ellipticity *θ*_MRW_ = (deg cm^2^ dmol^−1^) × 10^−6^.

### Thioflavin-T assay

Fluorescence experiments were performed using a Beckman fluorometer, using Quartz cells with a path length of 1 cm. The samples were freshly prepared with the dye ThT, and incubated in the cell while stirring at 37 °C. The solutions were prepared by adding NMR buffer (30 mM d-acetic acid, pH 5.3) to the dried peptide immediately prior to the measurements, holding the solution on ice all the time. A ThT stock solution was prepared in NMR buffer at a concentration of 25 mM. Final concentrations for the ThT experiment were 30 μM hIAPP and 10 μM ThT. The data were analyzed using the software Origin (OriginLab Corporation, Northampton, MA 01060, USA), and were fit assuming a sigmoidal behavior.

## Additional Information

**Accession codes:** The atomic coordinates and restraint files of the oxidized and amidated hIAPP are deposited in the PDB with accession code 5MGQ, chemical shifts at BMRB, entry 34069.

**How to cite this article**: Camargo, D. C. R. *et al*. The redox environment triggers conformational changes and aggregation of hIAPP in Type II Diabetes. *Sci. Rep.* **7**, 44041; doi: 10.1038/srep44041 (2017).

**Publisher's note:** Springer Nature remains neutral with regard to jurisdictional claims in published maps and institutional affiliations.

## Supplementary Material

Supplementary Information

## Figures and Tables

**Table 1:** Structural statistics of hIAPP (residues 1-37).

**NMR distance and dihedral constraints**	
Distance constraints	
Total NOE	542
Intra-residue	136
Inter-residue	406
Sequential (|*i* − *j*| = 1)	218
Medium-range (|*i* − *j*| < 4)	184
Long-range (|*i* − *j*| > 5)	4
Total dihedral angle restraints	20
*ϕ*	10
*ψ*	10
**Structure statistics**	
Violations (mean ± s.d.)	
Distance constraints (Å)	0.019 ± 0.002
Dihedral angle constraints (°)	0.767 ± 0.133
Max. dihedral angle violation (°)	3.02
Max. distance constraint violation (Å)	0.25
Deviations from idealized geometry	
Bond lengths (Å)	0.014 ± 0.001
Bond angles (°)	1.636 ± 0.043
Impropers (°)	1.789 ± 0.155
Average pairwise r.m.s. deviation* (Å)	
Heavy	0.78 ± 0.31
Backbone	0.27 ± 0.09
Ramachandran plot statistics (%)	
Residues in most favoured regions	68.0
Residues in additionally allowed regions	30.6
Residues in generously allowed regions	0.9
Residues in disallowed regions	0.5

*Pairwise r.m.s. deviation was calculated among 20 refined structures for residues 2–17.
