# Supplementary material for: The redox environment triggers conformational changes and aggregation of hIAPP in Type II Diabetes
Source: Sci Rep. 2017 Mar 13;7:44041. doi: 10.1038/srep44041 (PMC5347123; doi:10.1038/srep44041)
Supplement: Supplementary Information [file srep44041-s1.pdf]

Supplementary information for the manuscript:

The redox environment triggers conformational changes and aggregation of hIAPP in Type II Diabetes

Diana C. Rodriguez Camargo,[1,2] Konstantinos Tripsianes,[3] Katalin Buday,[1] Andras Franko,[1,4] Christoph Göbl,[1,2] Christoph Hartlmüller,[2] Riddhiman Sarkar,[1,2] Michaela Aichler,[1] Gabriele Mettenleiter,[1] Michael Schulz,[1] Annett Böddrich,[6] Christian Erck,[7] Henrik Martens,[7] Axel Karl Walch,[1] Tobias Madl,[1,2,5] Erich E. Wanker,[6] Marcus Conrad,[1] Martin Hrabě de Angelis,[1,4,8] and Bernd Reif[1,2]

[1] Helmholtz Zentrum München, Ingolstädter Landstr. 1, 85764 Neuherberg, Germany
[2] Munich Center for Integrated Protein Science (CIPS-M) at Department Chemie, Technische Universität München (TUM), Germany
[3] Central European Institute of Technology (CEITEC), Masaryk University, Kamenice 5, 62500 Brno, Czech Republic
[4] German Center for Diabetes Research (DZD e.V.), 85764 Neuherberg, Germany
[5] Institute of Molecular Biology & Biochemistry, Center of Molecular Medicine, Medical University of Graz, Austria
[6] Max-Delbrück-Center Berlin (MDC), Robert-Rössle-Str. 10, 13125 Berlin, Germany
[7] Synaptic Systems GmbH, Rudolf-Wissell-Straße 28, Göttingen, 37079 Germany
[8] Technische Universität München, Center of Life and Food Sciences Weihenstephan, 85354 Freising, Germany

Dot blot assay

For dot blot assays, IAPP monomer [HFIP-treated IAPP peptide dissolved in 20 mM Tris buffer (pH 7.4), 1 min sonicated] and fibril solutions were spotted onto a nitrocellulose membrane (AmershamTM ProtranTM 0.1 µm NC). After washing with 1x PBS (13.7 mM NaCl, 0.27 mM KCl, 1 mM Na_2HPO4, 0.2 mM KH_2PO_4, pH 7.4), the membrane was blocked for 30 min with 3% skim milk (Sigma-Aldrich) in 1xPBS containing 0.05% Tween 20 (PBS-T). Then the membrane was incubated over night with the primary IAPP antibody 91D7E8 diluted in 3% skim milk-PBS-T. Subsequently, the membrane was washed 3 times for 10 min in PBS-T and incubated with the secondary peroxidase-conjugated anti-mouse antibody for 1 h at room temperature. The membrane was washed 2 times for 5 min in PBS-T and 2 times for 10 min in PBS, and immunoreactive protein detected using ChemiGlow (Biozym). Chemiluminescence was measured with a FujiFilm LAS-3000; signals were quantified using the Aida image analysis software (Raytest, Germany). For amido black staining, nitrocellulose membranes containing spotted proteins were incubated for 1 min in 1x Amido black staining solution (Sigma Aldrich) and then incubated for 30 min in an aqueous solution containing 25% isopropanol and 10% acetic acid. Finally, images from the stained membranes were taken using the FujiFilm LAS-3000 system (Supporting Figure 1).

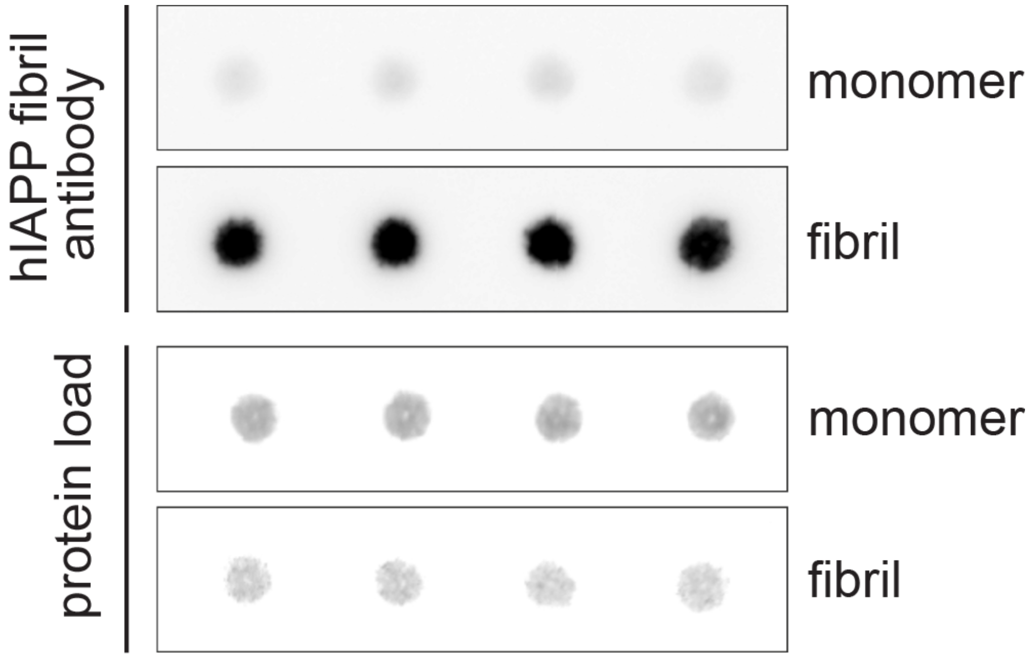

Supporting Figure 1. *IAPP antibody 91D7E8 specifically detects IAPP fibrils. Analysis of 1,1 µg IAPP monomer (Mon) and 1,1 µg IAPP fibrils (Fib) in quadruplicates on dotblots using the IAPP antibody 91D7E8. Amido black staining of IAPP monomer and fibril dotblots reveals an equal protein load.*

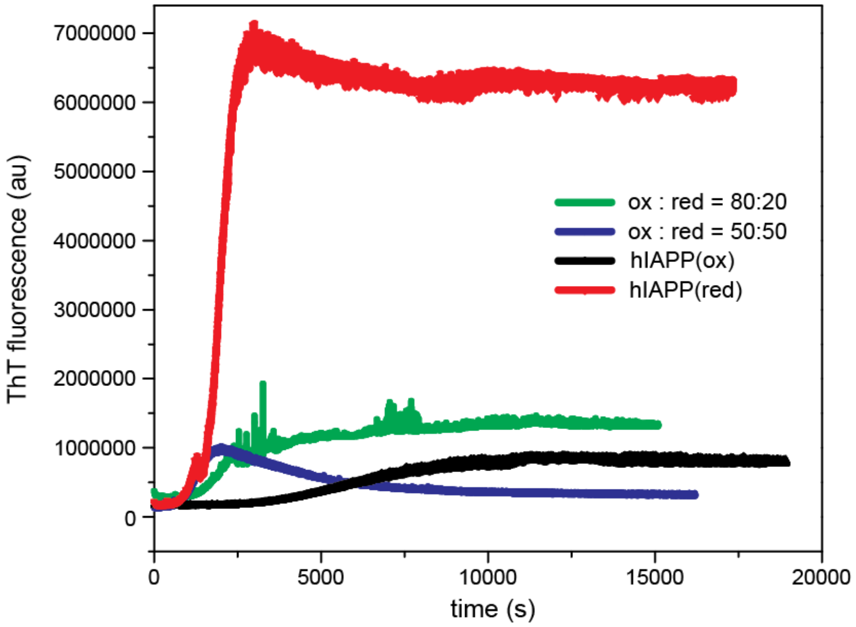

Supporting Figure 2. *Thioflavin T (ThT) assay using a solution of 35 μM of hIAPP in acetic acid, pH 5.3 and 30 °C. Experiments were performed by adding defined amounts of GSH and GSSG to yield varying percentages of reduced and oxidized hIAPP in solution. In contrast to Fig. 1A of the main manuscript, absolute ThT fluorescence intensites are represented.*

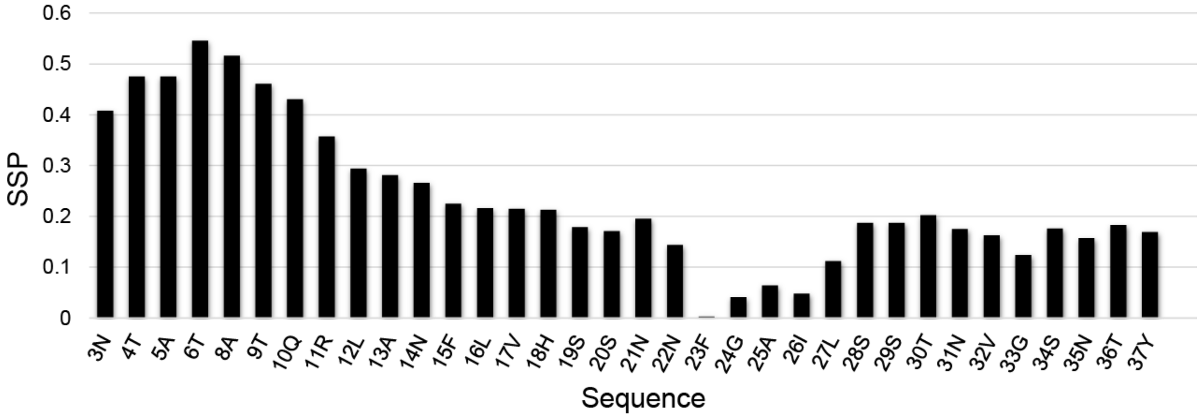

Supporting Figure 3. *Secondary Structure Propensity (SSP) of hIAPP^{ox} in aqueous buffer, pH 5.3. The SSP index has been calculated using the software package SPP developed by Forman-Kay and coworker.*[1]

RDC solution-state NMR experiments

Uniformly enriched ^{15}N hIAPP was freshly expressed and purified. The lyophilized powder was dissolved in NMR buffer (30 mM acetic acid, pH 5.3) to yield a 200 μM stock solution. The alignment medium was prepared by dissolving 15.6 μL pentaethylene glycol monooctyl ether (PEG, C8E5) in 250 μL of NMR buffer, and by subsequent addition of 8.25 μL octanol (Sigma-Aldrich). The solution was vigorously vortexed at 278 K. The medium yielded a D_2O deuterium doublet splitting of 25.5 Hz. The hIAPP solution was added to the liquid crystalline phase and diluted using NMR buffer to yield a final concentration of 100 μM for hIAPP and a C8E5-to-water ratio of 1.5% (w/v). The sample was measured at 278 K on a 600 MHz Av-III Bruker NMR spectrometer, equipped with gradient coils in the z-direction. The final sample yielded a stable D_2O doublet of 5.7 Hz after allowing for a period of stabilization of alignment monitored by 1-dimensional deuterium spectra. ^1H,^{15}N IPAP-HSQC[2] spectra were recorded using 1024 x 64 complex data points and 64 dummy scans to ensure temperature stability. The same parameters were applied to a sample, lacking the alignment medium to obtain a reference dataset. Spectra were processed and peak-picked using NMRPipe. The data were fit to the experimental solution structure by minimizing the difference between the experimental and calculated RDC values for each of the lowest 10 energy models.[3] Overlapping and non-resolved residues have been removed from the analysis. The structured part of the molecule (residue 1-20) was analyzed separately from the flexible tail (21-37) as shown in the Supporting Figure 4.

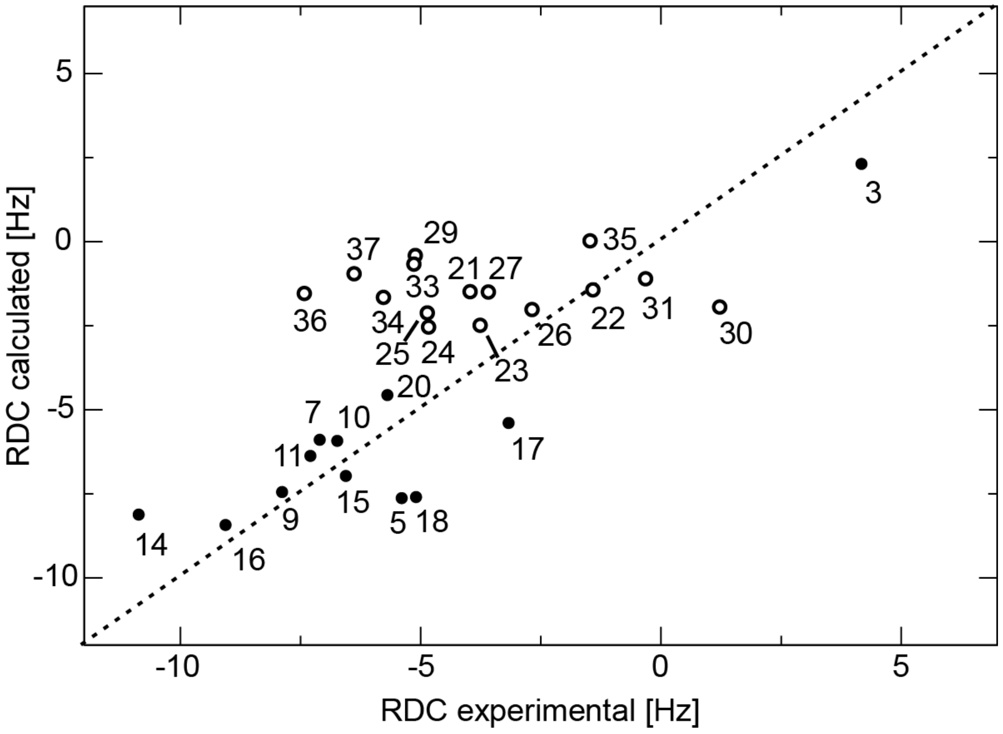

Supporting Figure 4. *Correlation between experimental and calculated RDCs under partial alignment with 1.5% PEG. Full circles represent data points from the helical N-terminus,*

open circles data points of the flexible C-terminus, respectively. Calculated RDC values correspond to the average of the lowest 10 energy conformations.

References

1. Marsh, J.A., Singh, V.K., Jia, Z. & Forman-Kay, J.D. Sensitivity of secondary structure propensities to sequence differences between α- and γ-synuclein: Implications for fibrillation. *Prot. Sci.* **15**, 2795-2804 (2006).
2. Ottiger, M., Delaglio, F. & Bax, A. Measurement of J and dipolar couplings from simplified two-dimensional NMR spectra. *J. Magn. Reson.* **131**, 373-378 (1998).
3. de Alba, E. & Tjandra, N. NMR dipolar couplings for the structure determination of biopolymers in solution. *Prog. NMR Spect.* **40**, 175-197 (2002).
